# The clinical impact of artemisinin resistance in Southeast Asia and the potential for future spread

**DOI:** 10.1093/femsre/fuw037

**Published:** 2017-01-01

**Authors:** Charles J. Woodrow, Nicholas J. White

**Affiliations:** Mahidol-Oxford Tropical Medicine Research Unit, Faculty of Tropical Medicine, Mahidol University, 420/6, Rajvithi Road, Bangkok 10400, Thailand

**Keywords:** artemisinin, resistance, malaria, ACT, southeast Asia, kelch

## Abstract

Artemisinins are the most rapidly acting of currently available antimalarial drugs. Artesunate has become the treatment of choice for severe malaria, and artemisinin-based combination therapies (ACTs) are the foundation of modern falciparum malaria treatment globally. Their safety and tolerability profile is excellent. Unfortunately, *Plasmodium falciparum* infections with mutations in the ‘K13’ gene, with reduced ring-stage susceptibility to artemisinins, and slow parasite clearance in patients treated with ACTs, are now widespread in Southeast Asia. We review clinical efficacy data from the region (2000–2015) that provides strong evidence that the loss of first-line ACTs in western Cambodia, first artesunate-mefloquine and then DHA-piperaquine, can be attributed primarily to K13 mutated parasites. The ring-stage activity of artemisinins is therefore critical for the sustained efficacy of ACTs; once it is lost, rapid selection of partner drug resistance and ACT failure are inevitable consequences. Consensus methods for monitoring artemisinin resistance are now available. Despite increased investment in regional control activities, ACTs are failing across an expanding area of the Greater Mekong subregion. Although multiple K13 mutations have arisen independently, successful multidrug-resistant parasite genotypes are taking over and threaten to spread to India and Africa. Stronger containment efforts and new approaches to sustaining long-term efficacy of antimalarial regimens are needed to prevent a global malaria emergency.

## Abstract (precis)

Artemisinin resistance in *Plasmodium falciparum* malaria is causing failure of artemisinin-based combination therapies across an expanding area of Southeast Asia, undermining control and elimination efforts. The potential global consequences can only be avoided by new approaches that ensure sustained efficacy for antimalarial regimens in malaria affected populations.

## Graphical abstract

**Graphical Abstract Figure.**
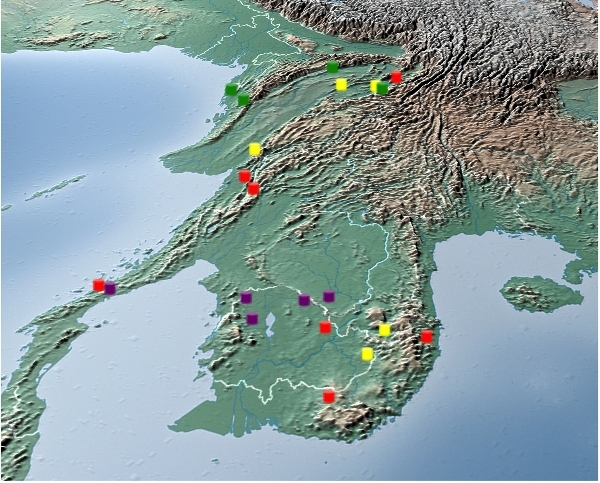
Artemisinin resistance in *Plasmodium falciparum* malaria is causing failure of artemisinin-based combination therapies across an expanding area of Southeast Asia, undermining control and elimination efforts. The potential global consequences can only be avoided by new approaches that ensure sustained efficacy for antimalarial regimens in malaria affected populations.

## INTRODUCTION

By the 1990s, *Plasmodium falciparum* malaria parasites resistant to multiple drugs (chloroquine, antifolates and mefloquine) were prevalent in Southeast Asia. The benefits of combinations were well established in infectious disease and cancer treatments, and had been used in the treatment of malaria since the discovery of plasmoquine (pamaquine) in the 1920s; chloroquine, amodiaquine, quinine and mefloquine had individually been combined with sulphadoxine-pyrimethamine. However, all had failed rapidly, unsurprisingly given pre-existing resistance to the components. Artemisinin-based combination therapies (ACTs) were introduced in direct response to this deteriorating situation. ACTs provided rapidly and reliably effective, well-tolerated antimalarial regimens with sustained efficacy. By 2006, ACTs were recommended as standard treatment for falciparum malaria worldwide. Their increasing deployment has been one of the main factors behind the reduction in malaria transmission in Asia (Peak *et al.*^2015^) and beyond (Bhatt *et al.*^2015^).

Unfortunately, over the last decade evidence has grown that artemisinin resistance has emerged and spread within Southeast Asia, first in western Cambodia but now across a widening area of the Greater Mekong Subregion (GMS). Rapid scientific advances in understanding of this problem have taken place within the last five years (Winzeler and Manary 2014; Fairhurst 2015; Tilley *et al.*^2016^). Here we review how and why artemisinin resistance has emerged in Southeast Asia, its clinical impact and what measures can be taken to prevent the problem from threatening malaria control globally.

## ARTEMISININ COMBINATION THERAPIES

All widely used artemisinin-containing treatments contain one of three artemisinin derivatives: artesunate, artemether or dihydroartemisinin (DHA). Oral artesunate and artemether are active themselves but are also converted by blood esterases and hepatic cytochrome P450 enzymes respectively to DHA, which provides the majority of their activity. Artemisinins are rapidly absorbed and have a short elimination half-life of approximately 1 h, but this is sufficient time for them to exert maximal effects against both asexual stages and immature gametocytes.

Artemisinins have two distinctive pharmacodynamic properties that determine how they are best used. First, they kill both young rings and more mature trophozoites rapidly, an action important for life-saving efficacy in severe disease as well as cure (Dondorp *et al.*^2005^, ^2010^). Clearance of drug-affected ring stage parasites involves pitting in the spleen with removal of the intraerythrocytic parasite and return of the once-infected red cell to the circulation (Chotivanich *et al.*^2000^; Buffet *et al.*^2006^). Because of their ring-stage activity, parasite clearance rates following treatment with artemisinins are much more rapid than with other antimalarials such as quinine (White 2004). Second, a small subpopulation of artemisinin-treated rings enters a state of quiescence or dormancy rather than being killed outright, resuming growth only after a period of days to weeks (Teuscher *et al.*^2010^; Witkowski *et al.*^2010^). This property is thought to underlie the approximate 10% failure rates observed when artemisinins are administered as monotherapies, even with directly observed 7-day treatments (Li *et al.*^1984^; Price *et al.*1998a,b). The rapid initial rates of parasitaemia reduction and the full *in vitro* susceptibility of recrudescent isolates argue against this resulting from inadequate killing of all parasites.

For these reasons, artemisinins are best suited to use in partnership with a more slowly eliminated, longer acting drug; the artemisinin component kills the bulk of the malaria parasites within a few days, while the partner drug persists for long enough to kill the relatively few that remain (White 2004). By the 1990s, with multidrug-resistant *Plasmodium falciparum* widespread in Thailand and neighbouring areas, and no effective alternative drugs available, artesunate was combined with the failing mefloquine (Nosten *et al.*^1994^). The combination of mefloquine with three daily doses of artesunate improved cure rates to satisfactory levels (more than 95%) and provided an effective regimen that reduced the incidence of falciparum malaria along the Thailand–Myanmar border substantially (Carrara *et al.*^2009^); indeed the remaining parasites in the area became more sensitive to mefloquine (Nosten *et al.*^2000^). In effect the addition of the artemisinin derivative had reversed the decline in mefloquine efficacy.

This approach was then extended to other areas, involving different artemisinin derivatives and partner drugs (Adjuik *et al.*^2004^). Several coformulated ACTs are now deployed including artemether-lumefantrine, DHA-piperaquine, artesunate-amodiaquine and artesunate-mefloquine. Outside Southeast Asia, these treatments provide uniformly excellent cure rates (well above the minimum acceptable threshold of 90%). ACTs have been recommended for falciparum malaria everywhere by WHO since 2006. New ACTs have also been developed, for example, artesunate-pyronaridine (Rueangweerayut *et al.*^2012^). Artesunate with sulphadoxine-pyrimethamine as the partner drug has also provided acceptable efficacy in areas where resistance to sulphadoxine and pyrimethamine has never reached high levels, although in contrast to mefloquine there has not been evidence of actual improvement in terms of resistance to the partner components.

## FALLING ARTEMISININ SUSCEPTIBILITY IN CAMBODIA AND THAILAND

### Therapeutic efficacy studies

Artemisinins have been readily available in Cambodia and Vietnam for well over 20 years. Sporadic reports of poor therapeutic responses to artemisinins have occurred throughout this period, but were usually explained by counterfeit (falsified) medicines. Evidence that *Plasmodium falciparum* might not be responding to ACTs as before began to emerge in therapeutic efficacy studies undertaken on either side of the Thai–Cambodia border; in 2002, in Pailin (western Cambodia) the day 28 failure rate for artesunate plus mefloquine was 14%, and in a subsequent 2004 study when follow-up was extended to 42 days the failure rate was 21% (Denis *et al.*2006b). In nearby Battambang Province, unsatisfactory cure rates were also observed with artemether-lumefantrine (Denis *et al.*2006a). Inadequate day 28 cure rates for artesunate (given for 2 rather than 3 days) plus mefloquine were also observed on the Thai side of the border in Trat Province in 2003 (Vijaykadga *et al.*^2006^). Initially, this was ascribed to partner drug resistance.

A subsequent study in southern Cambodia with a 42-day follow-up period also documented unsatisfactory cure rates with artesunate plus mefloquine (Rogers *et al.*^2009^). Parasite clearance was slower than expected; 47% of patients remained parasite positive by blood smear 2 days after the start of treatment (compared to 10% in previous studies) and 11.3% of patients were still positive at day 3, a finding that was associated with recrudescence. This provided the clearest evidence to date that artemisinin resistance might be a contributing factor to the failure of an ACT.

### Focused artemisinin sensitivity trials

A series of clinical efficacy studies was designed to study the problem of artemisinin resistance in more detail than provided by standard therapeutic efficacy studies (the ARC Projects) (Fairhurst *et al.*^2012^). Artesunate was administered as monotherapy, with frequent quantitation of parasitaemia to determine rates of parasite clearance without risk of confounding by partner drug (Stepniewska *et al.*^2010^). These studies confirmed that despite adequate drug levels, parasite clearance rates were twice as slow in the western Cambodian provinces of Battambang (Noedl *et al.*^2008^, ^2010^), Pailin (Dondorp *et al.*^2009^) and Pursat (Amaratunga *et al.*^2012^) than on the Thai–Myanmar border, although longitudinal studies along this border also indicated slow parasite clearance (Carrara *et al.*^2009^; Phyo *et al.*^2012^). Slow clearing infections were subsequently found to be common across mainland Southeast Asia both in individual studies (Hien *et al.*^2012^; Kyaw *et al.*^2013^) and the Tracking Resistance to Artemisinin Collaboration (TRAC) study that employed a common protocol across 10 sites in the region (Ashley *et al.*^2014^).

Studies during this period also showed that increasing or splitting the individual daily dose of artemisinin derivative did not increase the rate of parasite clearance (Bethell *et al.*^2011^; Das *et al.*^2013^), although a daily artesunate dose of 6 mg kg^−1^ risked haematological toxicity (Bethell *et al.*^2011^).

### Modified *in vitro* assays

Despite the substantial reductions in clinical response to artemisinins observed in falciparum malaria, the *in vitro* concentrations of artesunate resulting in 50% growth inhibition in a standard 48-h exposure assay were not generally high and did not predict slow parasite clearance or ACT failure. The proposed explanation for this, also supported by modelling studies, was that artemisinin resistance affected predominantly ring-stage parasites (Dondorp *et al.*^2009^; Saralamba *et al.*^2011^). However, it is worth noting that a subset of HRP2-based *in vitro* studies, where signal is partly generated in the ring stages, did report higher IC50 values for DHA in cases with poor clinical responses (Noedl *et al.*^2010^) as well as rising values over time (Tyner *et al.*^2012^). How to assess the *in vitro* artemisinin susceptibility of ring stages became an important area of study. Ring-stage specific assays of *P. falciparum in vitro* susceptibility had been described for more than two decades in an experimental context (Geary, Divo and Jensen 1989; ter Kuile *et al.*^1993^; Alin and Bjorkman 1994; Skinner *et al.*^1996^). Witkowski *et al.* (2013a,b) described a ring-stage survival assay (RSA) suitable for practical use. The RSA broadly mimics clinical exposure, with tightly synchronised rings (0–3 h of the 48 h cycle) exposed to a 6-h ‘pulse’ of 700 nM DHA, which is then removed by washing, following which parasites are left to grow into the next cycle before microscopy assessment. Based on a 1% parasite survival rate as cut-off, the RSA confirmed that ring stages of *P. falciparum* isolates from western Cambodia were highly resistant to DHA, independently of host variables (Witkowski et al. 2013a,b). Pulse assays of a similar design were subsequently developed by other groups (Dogovski *et al.*^2015^; Hott *et al.*^2015^). An alternative simple method assessing maturation up to the trophozoite stage in *ex vivo* isolates as in a standard micro test also correlates with clinical responses (Chotivanich *et al.*^2014^).

### The identification of ‘K13’

Slow parasite clearance in the clinical studies described above provided the signal for a series of genotype–phenotype association studies. The clinical parasitological response was clearly heritable (Anderson *et al.*^2010^; Amaratunga *et al.*^2012^; Takala-Harrison *et al.*^2013^) indicating a genetic basis, with reduced artemisinin susceptibility associated with multiple founder populations (Miotto *et al.*^2013^). Genome-wide studies detected a region on *P. falciparum* chromosome 13 that was strongly associated with slow *in vivo* parasite clearance (Cheeseman *et al.*^2012^; Takala-Harrison *et al.*^2013^).

The pivotal breakthrough came at the end of 2013 when parasites cultured for five years under intermittent artemisinin pressure were sequenced (Witkowski *et al.*^2010^; Ariey *et al.*^2014^) and a mutation found close to the chromosome 13 region identified in the genetic association studies (Cheeseman *et al.*^2012^; Takala-Harrison *et al.*^2013^). The mutated gene in question encodes a protein containing a ‘kelch’ motif (Ariey *et al.*^2014^) and is now generally known as ‘K13’. A high proportion of Cambodian isolates had mutations in K13's ‘propeller’ region, a six-bladed structure that mediates interactions between kelch proteins and other macromolecules. Although there were a wide range of K13 mutations, in each isolate generally only one mutation was present. The prevalence of propeller mutations clearly correlated with parasite survival rates *in vitro* as well as the proportion of patients with day 3 parasitaemia positivity in therapeutic efficacy studies (Ariey *et al.*^2014^). Soon after, the multicentre TRAC study confirmed that slowly clearing infections (parasite clearance half-life >5 h) were strongly associated with K13 propeller mutations across Southeast Asia (Ashley *et al.*^2014^). In western Cambodia, the C580Y mutation was by far the most prevalent, being almost at fixation in several sites, suggesting that this mutation is the most ‘successful’ over time. Elsewhere other mutations were more common. The causative role of C580Y and certain other K13 mutations in mediating reduced artemisinin susceptibility has since been confirmed by experimental manipulation of the genetic locus (Ghorbal *et al.*^2014^; Straimer *et al.*^2015^).

Identifying K13 provided a major stimulus to the field by increasing understanding of resistance and providing control programmes and researchers with a practical tool for monitoring the extent of artemisinin resistance (Fairhurst 2015) that complements the phenotypic methods already discussed.

## IS IT ARTEMISININ RESISTANCE?

Should the reduced artemisinin susceptibility developing in Southeast Asia be referred to as artemisinin ‘resistance’? In 1967, WHO defined ‘drug resistance’ as the ability of a parasite strain to survive or multiply despite the administration and absorption of a drug given in doses equal to or higher than those usually recommended but within the tolerance of the subject (World Health Organization 1967). The definition is logically based upon *in vivo* efficacy (Basco 2007) since this is what matters in terms of managing the individual patient, as well as the problem of drug resistance in a population over the long term. We review the epidemiological evidence that the reduced artemisinin susceptibility associated with K13 mutation has led to partner drug resistance, and failure of ACTs.

**Figure 1.**
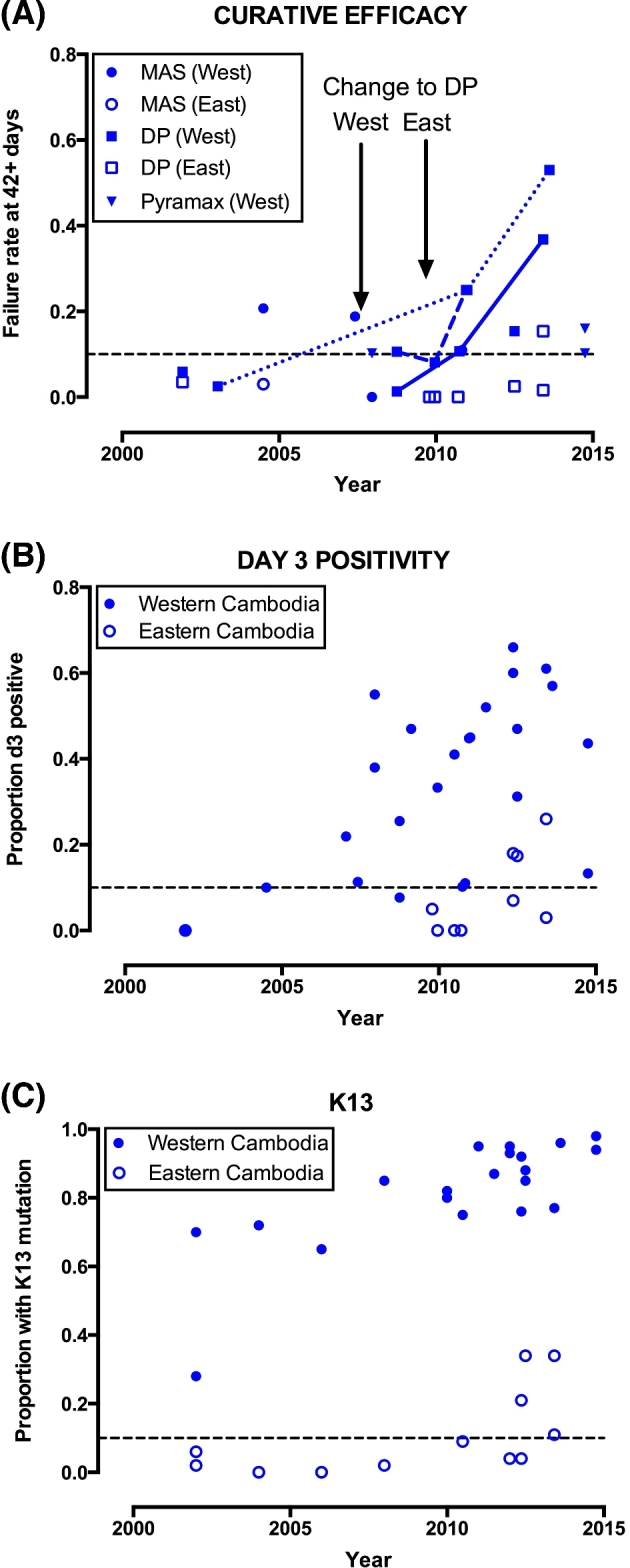
Longitudinal trends in cure rate (**A**), day 3 positivity (**B**) and K13 mutation prevalence (**C**), Cambodia, 2000–2015. In section A (cure rate), connecting lines are drawn for serial studies undertaken in Pursat (normal line), Pailin (dashed) and Oddar Meanchey (dotted) provinces. MAS = artesunate plus mefloquine, DP = DHA-piperaquine, Pyramax = artesunate-pyronaridine. Locations are stratified into western and eastern provinces according to their position with respect to the capital Phnom Penh, in line with previous work (Leang *et al.*^2015^). The midpoint of patient recruitment is used as the time point for each study, or half-way through the year if months were not stated. Only studies with *n* = 20 or more are included. For references see Additional File (Supporting Information).

To illustrate the time course of increasing ACT failures, slowing parasite clearance times and prevalence of K13 mutation, we have summarised graphically how these measures have varied over time in Cambodia (Fig. 1). This shows that by 2001 K13 propeller mutations were highly prevalent in western Cambodia and slowly clearing parasites (based on day 3 positivity) were evident by 2004. This suggests that the declining efficacy of ACTs observed from 2004 onwards (previously attributed to partner drug resistance and host pharmacological factors) is also likely to reflect loss of ring-stage artemisinin activity. For example, artesunate plus mefloquine showed inadequate efficacy in Pailin in 2004; mefloquine resistance was clearly present (Alker *et al.*^2007^) but this alone is unlikely to have been the sole explanation for the problem, since at the Thai–Myanmar border efficacy of this ACT was maintained for many years after mefloquine resistance reached high levels (Price *et al.*^2004^; Carrara *et al.*^2009^). Similarly the failures observed with artemether-lumefantrine were probably not due to poor lumefantrine absorption alone (Denis *et al.*2006a) as efficacy remained unsatisfactory even with fatty food supplementation. Pharmacological explanations for slow parasite clearance were also ruled out in the detailed artemisinin sensitivity studies that showed consistent drug absorption (Dondorp *et al.*^2009^). Although immunity can affect parasite clearance kinetics (Borrmann *et al.*^2011^; WWARN Parasite Clearance Study Group 2015; Simpson, McCaw and Fowkes 2016), the magnitude of the changes in clearance rate observed in Southeast Asia, as well as the lack of an age effect in large clearance rate studies (Ashley *et al.*^2014^), argues strongly against a major role for reduced immunity as a determinant of declining ACT efficacy.

The series of therapeutic efficacy studies documenting the efficacy of DHA-piperaquine after its introduction as treatment policy for falciparum malaria in western Cambodia in 2008 sheds further light on this question. DHA-piperaquine had been used only briefly in the country, and previous studies had shown satisfactory efficacy (Denis *et al.*^2002^; Janssens *et al.*^2007^). But even at the time of policy change (2008), the efficacy of DHA-piperaquine in western Cambodia was of borderline acceptability (Leang *et al.*^2013^), and it worsened rapidly thereafter in all locations studied (Fig. 1A) (Leang *et al.*^2013^, ^2015^; Lon *et al.*^2014^; Spring *et al.*^2015^; Amaratunga *et al.*^2016^). For example, in Oddar Meanchey Province, northwest Cambodia, DHA-piperaquine gave a 63-day efficacy of 97.5% in 2002–2003 (Janssens *et al.*^2007^), but by 2013 more than half of the treated patients suffered a recrudescence (Saunders, Vanachayangkul and Lon 2014; Spring *et al.*^2015^). The efficacy of DHA-piperaquine is now falling in other regions far from where initial concerns were raised (Amaratunga *et al.*^2016^). Treatment policy in Cambodia has now been forced to revert to artesunate-mefloquine.

When ACTs were first introduced, the theory was that the ring-stage action of artemisinins was critical to the rapid killing of circulating parasites. Whether this ring-stage activity was required for patient cure and long-term success of the combination was not known. It is now evident that the loss of ring-stage activity of artemisinins associated with K13 mutation causes a substantially greater proportion of parasites to survive the initial phase of treatment, and over time this leads to partner drug resistance and a decline in the efficacy of ACTs when deployed at scale. Since increasing the dose of artemisinin derivative to counter this problem is not tolerated clinically, and is ineffective, it is appropriate to call this ‘resistance’, and this designation is now widely accepted, for example, in the regular updates on artemisinin resistance issued by the WHO (World Health Organization 2015).

## HOW DO K13 MUTATIONS CAUSE ARTEMISININ RESISTANCE?

At the biochemical level, the range of mutations in K13, and the fact that a single mutation is sufficient to cause artemisinin resistance, suggests that the mutations observed in Cambodian isolates mediate loss of function of the K13 protein. Loss-of-function mutations in kelch domains have been found in a range of human cancers as well as inherited metabolic syndromes (Padmanabhan *et al.*^2006^; Boyden *et al.*^2012^). A large body of research has documented how human kelch-containing proteins serve as adaptors that bring substrates into ubiquitination complexes. For example, the protein keap1 binds the transcription factor nrf2, leading to its ubiquitination and subsequent degradation, and loss-of-function mutations in keap1 lead to increased levels of nrf2 and constitutive activation of antioxidant pathways (Hayes and McMahon 2006).

**Figure 2.**
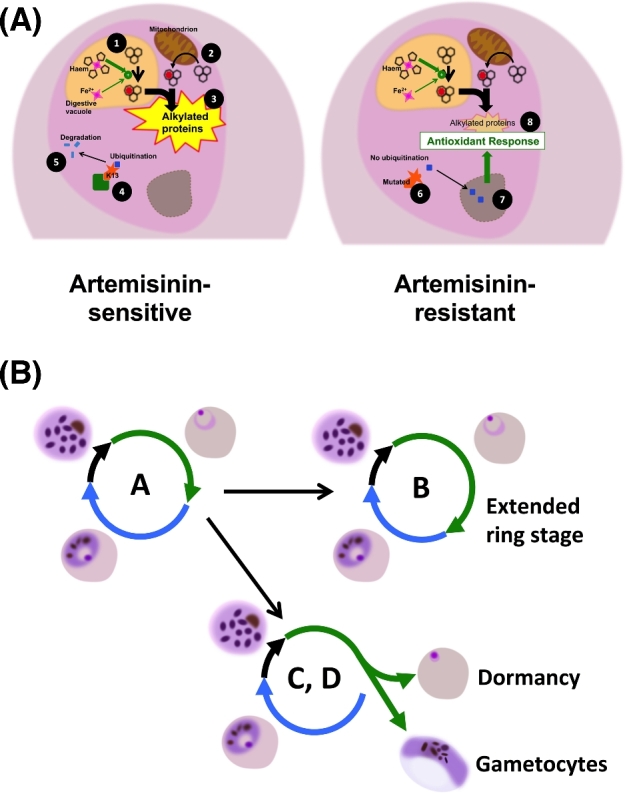
Proposed mechanisms of artemisinin resistance. (**A)** Relevant biochemical pathways. In ring-stage parasites, artemisinin is primarily activated by haem produced in the process of haemoglobin digestion (1) although haem biosynthesis in mitochondria may also contribute (2). Activated artemisinins alkylate nearby proteins in an indiscriminate manner leading to cell death (3). In artemisinin-sensitive parasites, a transcriptional factor with potential to upregulate protein turnover and oxidative damage responses is bound via the K13 adaptor (4) leading to its ubiquitination and proteolysis (5). K13 mutation disrupts this binding (6) allowing the factor to enter the nucleus (7) with upregulation of a range of transcriptional responses that can mitigate the downstream consequences of artemisinins (8). (**B)** Proposed phenotypes associated with artemisinin resistance. The overall length and proportion of time spent in each stage appears relatively fixed in a given strain (A). By extending their ring-stage (B), parasites increase the period of reduced vulnerability to artemisinins. An alternative is to increase the proportion of parasites entering dormancy (C), a natural phenomenon observed in all parasite strains that allows escape from relatively short duration artemisinin exposures in patient treatments. Finally, increasing the proportion of parasites that differentiate into gametocytes (D) at a given timepoint could improve chances of transmission before treatment is administered.

Artemisinin resistance indeed appears to operate through widespread changes in transcription (Fig. 2A). Artemisinin-resistant parasites from the TRAC study were found to have profound transcriptional alterations with an unfolded protein response that may be constitutively (as opposed to intermittently) activated, increasing the capacity of parasites to repair or quickly degrade proteins (or other cellular components) damaged by artemisinin exposure (Mok *et al.*^2015^). Separate studies involving tight synchronisation of culture-adapted lines also support the hypothesis that an enhanced cellular stress response underlies resistance, with K13-mutant parasites exhibiting decreased artemisinin sensitivity across a full third of the parasite cycle (Dogovski *et al.*^2015^). The development of resistance via mitigation of the damage inflicted by artemisinins would also fit with artemisinins exerting their action via pleiotropic effects (Cobbold *et al.*^2016^) involving alkylation (Wang *et al.*2015a; Ismail *et al.*^2016^) after activation by haem derived from haemoglobin digestion (Klonis *et al.*^2011^, ^2013^; Xie *et al.*^2016^).

Recent *in vitro* studies have probed how artemisinin resistance operates at the level of the parasite life cycle (Fig. 2B). In the transcriptomic studies described above, resistant parasites exhibited decelerated progression through the first part of the asexual intraerythrocytic development cycle, potentially reducing artemisinin activation or extending the period for repair of damaged proteins (Mok *et al.*^2015^). Altered patterns of development have also been observed in culture-adapted isolates, with the proposal that this reduces exposure to artemisinin at the most susceptible stage of development in erythrocytes (trophozoites) and increases exposure in the more resistant stage (rings) (Hott *et al.*^2015^). However, among isolates with different K13 sequences, there was no simple correlative relationship with the length of the ring-stage or intraerythrocytic cycle (Dogovski *et al.*^2015^).

Along with increased survival in the relatively short term after a pulse of artemisinin treatment in the RSA (see above), artemisinin resistance may also enhance the natural propensity for parasites to persist via quiescence (dormancy) after artemisinin exposure, allowing subsequent recovery days or weeks later. In the artemisinin-pressured line in which the K13 mutation was first described, as well as culture-adapted field isolates, a higher proportion of ring stages enter developmental arrest and subsequently exit quiescence in response to artemisinins, as well as other drugs (Witkowski *et al.*2013b; Menard *et al.*^2015^). However, independent work on culture-adapted artemisinin-resistant field isolates, using a series of 6-h pulses of DHA, has not suggested a straightforward relationship between slow parasite clearance (or K13 mutation) and altered patterns of recovery after drug exposure (Hott *et al.*^2015^). Furthermore, dormancy cannot explain the majority of the delayed parasite clearance profiles observed *in vivo*.

In the TRAC study, the incidence of pre-treatment and post-treatment gametocytaemia was higher among patients with slow parasite clearance (Ashley *et al.*^2014^). Increased gametocytogenesis could increase the probability of transmission, and thus selection. More information on the transmissibility of artemisinin-resistant *Plasmodium falciparum* is needed.

A distinct proposition is that K13 mutations mediate artemisinin resistance via limiting their effects on specific ring-stage targets. Evidence has recently been presented that *P. falciparum* phosphatidylinositol-3-kinase (PfPI3K) is a specific target of artemisinins and that its levels are increased in parasites with K13 mutations (Mbengue *et al.*^2015^). It is worth noting that a previously proposed target, PfATP6, is not involved in artemisinin resistance (Cheeseman *et al.*^2012^; Miao *et al.*^2013^; Miotto *et al.*^2013^, ^2015^) and indeed no longer appears relevant to artemisinin action (David-Bosne *et al.*^2016^).

## DETERMINING THE EXTENT OF ARTEMISININ RESISTANCE

The extent of artemisinin resistance in *Plasmodium falciparum* can be assessed by a range of phenotypic and genotypic methods (Table 1). Each method has practical and theoretical pros and cons, and the most appropriate choice in a given situation has to take into account these issues.

**Table 1.** Pros and cons of five markers of artemisinin resistance.

	Therapeutic efficacy of ACT	Proportion of cases microscopy positive at day 3	Parasite clearance half-life	Ring-stage survival assay (RSA)	K13 sequencing
Influence on antimalarial policy	Direct	Indirect	Indirect	Indirect	Indirect
Confounded by partner drug	Yes	Yes	In theory^a^	No	No
Confounded by starting parasitaemia	Yes	Yes	Slightly^b^	No	No
Level of assessment	Population	Population	Individual	Individual	Individual
Convenient for patient	Requires at least 42 days follow-up	Minimal follow-up required	Requires frequent sampling for 2–3 days	Yes	Yes
Specific resources required	PCR differentiation for recurrences	None	Inpatient stay, accurate parasite quantification	Expertise, culture facilities	Access to sequencing

^a^No direct evidence, but theoretically possible given the influence of partner drug on day 3 positivity (Stepniewska *et al.*^2010^).

^b^In areas of artemisinin resistance, high parasitaemias were associated with slightly longer PC_1/2_ (by 5.2% per 10-fold increase) (WWARN Parasite Clearance Study Group 2015).

### Phenotypic assessment

Phenotyping of parasites is essential for monitoring artemisinin resistance. *In vitro* assessments remain important, and the RSA has been adapted successfully to allow flow cytometric output, bringing greater efficiency and objectivity (Amaratunga, Neal and Fairhurst 2014). However, the RSA requires resources and expertise in terms of synchronisation and culture adaptation that are rarely available in laboratories undertaking routine monitoring. Nevertheless the RSA or other validated ring stage susceptibility assessments remain critical for the assessment of phenotypes after transfection or genetic crosses.

Detailed parasite clearance studies provide a quantitative signal for individual infections. A standardised method for the assessment of parasite clearance has been developed that produces a parasite clearance half-life (PC_1/2_). Importantly, this method accounts for differences in parasitaemias and parasite stage distributions (and thus the variable lag phase in the post-treatment fall in parasitaemia) (Flegg *et al.*^2011^). However, measuring the rate of parasite clearance accurately can only be undertaken with a relatively high starting parasitaemia (more than 10 000 parasites μl^−1^) and requires measurement of parasitaemia every 6 to 8 h in the early phase (Flegg *et al.*^2013^; WWARN Parasite Clearance Study Group 2015), also making the approach resource intensive. Sensitive PCR detection allows a broader range of density quantitation but often reveals the dormant subpopulation, and may be confounded by gametocytaemia, and so needs to be validated in the measurement of parasite clearance rates.

The proportion of patients who are slide positive for malaria parasites on day 3 is much easier to measure and is reported widely. It does provide some useful information, although it is strongly influenced by starting parasitaemia and partner drug (Stepniewska *et al.*^2010^; Bethell *et al.*^2011^; WWARN ACT Africa Baseline Study Group 2015). If the day 3 positivity rate is less than 3%, resistance can be ruled out but higher values do not necessarily rule it in. Rather they act as an indication for further more detailed *in vivo* and *in vitro* studies (Stepniewska *et al.*^2010^). In practice a threshold of 10% has been useful in Southeast Asia in terms of identifying areas for more definitive clinical and laboratory studies (White *et al.*^2015^). In Africa, a 5% threshold provides a more sensitive benchmark for detecting delayed parasite clearance at an early stage (WWARN ACT Africa Baseline Study Group 2015).

**Figure 3.**
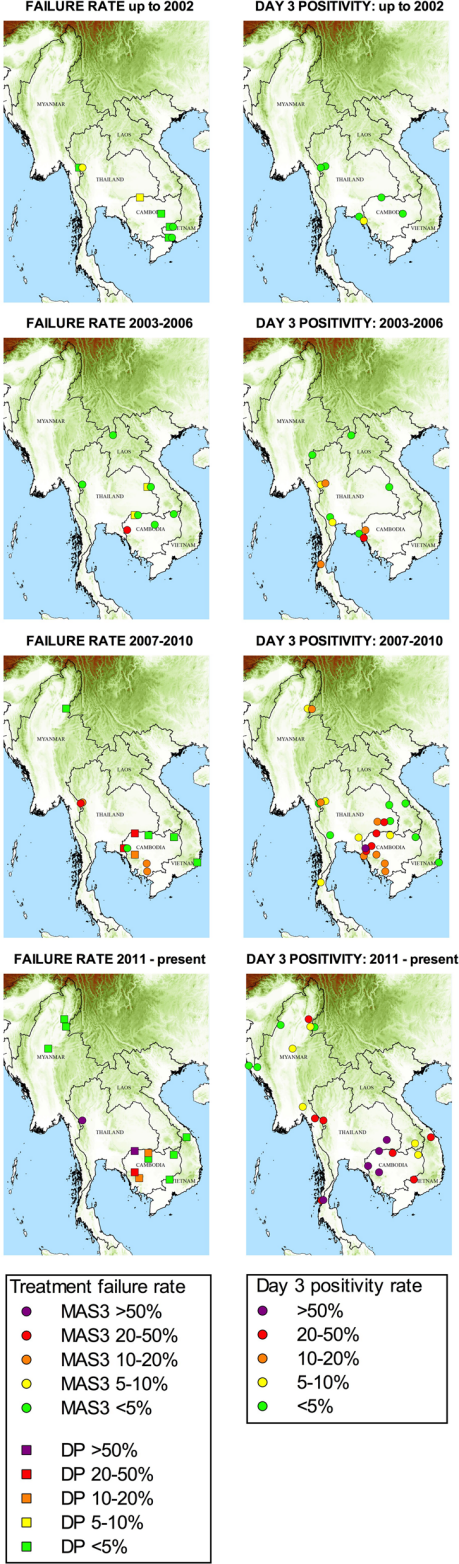
The extent of ACT failure (in studies with at least 42 days of follow-up) and day 3 positivity across four time periods from 2000 to the present. If two studies were available in one location for the same time period, the higher failure rate is shown. MAS3: artesunate plus mefloquine; DP: DHA-piperaquine. For references, see Additional File (Supporting Information).

We have plotted the rates of day 3 positivity after an artemisinin-containing regimen, and cure rates for DHA-piperaquine and artesunate plus mefloquine, for all studies undertaken in Southeast Asia since 2000, stratifying the data into four time periods (Fig. 3). These phenotypic studies confirm that artemisinin resistance clearly extends well beyond western Cambodia. Day 3 positivity rates in eastern Thailand have been elevated for at least five years (Satimai *et al.*^2012^). Longitudinal studies from the Thai–Myanmar border provide strong evidence for steadily increasing artemisinin resistance over the past decade (Phyo *et al.*^2012^), culminating in the loss of artesunate-mefloquine efficacy in a manner similar to that observed approximately five years earlier in western Cambodia (Na-Bangchang *et al.*^2010^; Carrara *et al.*^2013^). *In vivo* artemisinin sensitivity studies show that slow parasite clearance is also prevalent in southern Vietnam (Hien *et al.*^2012^), southern (Kyaw *et al.*^2013^) and central Myanmar (Ashley *et al.*^2014^) and the Myanmar–China border (Huang *et al.*^2015^). Slow clearance has also been documented in therapeutic ACT efficacy studies in a distinct location in Vietnam (Thriemer *et al.*^2014^) and in eastern (Nyunt *et al.*^2015^) and northern (Tun *et al.*^2016^) Myanmar. So far, there is no evidence of ACT failure inside Myanmar, but there have been very few efficacy studies with at least 42-day follow-up published in the last decade (Smithuis *et al.*^2010^; Tun *et al.*^2016^).

Importantly, there is no evidence that artemisinin resistance has worsened further in recent years in western Cambodia (WWARN Parasite Clearance Study Group 2015), where the C580Y mutation is at near fixation. But this should not be an excuse for complacency. DHA-piperaquine has been lost across much of Cambodia (Amaratunga *et al.*^2016^). Given what has gone before, it is reasonable to predict that ACT efficacy will fall in provinces that lie across the border in Thailand, Laos and Vietnam. Uncontained partner drug resistance with rapidly rising treatment failure rates will place strong selective pressure for even higher levels of artemisinin resistance.

### Genotypic surveys

Given its central role in artemisinin resistance, K13 can potentially serve as a standardised molecular marker allowing rapid assessment of the level of artemisinin resistance, particularly in remote locations where phenotypic studies are challenging (Roper *et al.*^2014^; Tun *et al.*^2015^). K13-based surveys have documented the presence of K13 mutations at high prevalence near the Myanmar–India border (Tun *et al.*^2015^) and in 93% of isolates (mostly C580Y) in a recent outbreak in northeast Thailand (Imwong *et al.*^2015^). One recent report has found K13 C580Y to be present in around 5% of isolates in a study from Guyana, although more information is needed on the clinical epidemiology of these parasites (Chenet *et al.*^2016^). Retrospective studies have also proven useful in understanding the development of artemisinin resistance based on historical samples (Ouattara *et al.*^2015^; Talundzic *et al.*^2015^; Putaporntip *et al.*^2016^).

In the original culture experiment which identified K13 as the cause of artemisinin resistance, a single K13 mutation in a parasite strain from Tanzania was associated with a substantial reduction in *in vitro* susceptibility (Ariey *et al.*^2014^). Not all K13 mutations are associated with artemisinin resistance—for example, the most common mutation, A578S, is typically found in 1% of isolates or less everywhere, is not associated with slow parasite clearance and in gene editing experiments does not confer artemisinin resistance *in vitro* (Ouattara *et al.*^2015^; Maiga-Ascofare and May 2016; Menard *et al.*^2016^). Surveys have so far provided reassuring evidence that K13 mutations have not reached high prevalences outside Southeast Asia (Menard *et al.*^2016^). K13 mutations are found in Africa, but only at low prevalence consistent with background variation rather than selection (Kamau *et al.*^2015^; Taylor *et al.*^2015^; MalariaGEN Plasmodium falciparum Community Project 2016; Menard *et al.*^2016^).

An important factor in the emergence of artemisinin resistance via selection of K13 mutations is the parasite genetic background. In the largest genome-wide association study undertaken to date, involving more than 1000 samples, mostly obtained in Southeast Asia, K13 produced the strongest signal, but polymorphisms in a number of other genes were also associated with slow clearance (Miotto *et al.*^2015^). These mutations collectively form a genetic ‘backbone’, common in much of mainland Southeast Asia, on which K13 mutations arise independently (Takala-Harrison *et al.*^2015^; Miotto *et al.*^2015^). The backbone mutations may compensate for the reduced fitness brought about by mutation of the highly conserved K13 protein, or boost the level of artemisinin resistance; they may also reflect resistance to previously used drugs. Studies on laboratory isolates and transfected lines confirm that the genetic background influences the degree of artemisinin susceptibility in ring-stage assays (Klonis *et al.*^2013^; Straimer *et al.*^2015^). The absence of a favourable genetic background may explain the lack of selection of K13 mutations in Africa to date.

**Figure 4.**
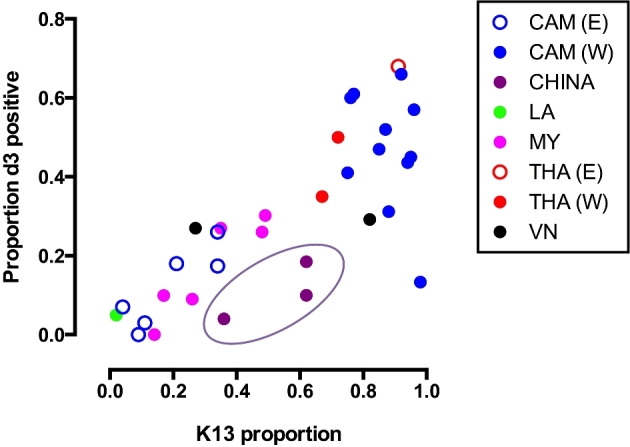
The relationship between the proportion of parasites with K13 propeller mutations and day 3 positivity in clinical studies in Southeast Asia. The oval ring indicates data from Yunnan Province, China in which the F446I K13 mutation was the most common. Only studies with *n* = 20 or more are included. For references see Additional File (Supporting Information).

There is an interplay between K13 mutation, genetic background and artemisinin susceptibility phenotype, and monitoring the extent of artemisinin resistance may require more than one approach. The quantitative relationship between K13 mutations that are common in western Cambodia and artemisinin resistance appears robust, and recent studies from the Thai–Myanmar border confirm the decisive role of K13 mutation in the loss of artesunate-mefloquine efficacy (Phyo *et al.*2016a). However, in other regions, and for other K13 mutations, this relationship is still being explored. A wide variety of different propeller mutations have now been found—more than 100 to date (Fairhurst 2015)—but few have been correlated with phenotype in significant numbers and shown to be clearly associated with resistance (World Health Organization 2015). We have examined the prevalence of K13 mutations in all studies from Southeast Asia that have reported day 3 positivity after any artemisinin-containing treatment (Fig. 4). Although this analysis is subject to the confounding effect of different starting parasitaemias and partner drugs on early clearance, it does suggest that the relationship between the prevalence of K13 mutation and slow clearance is not a constant one.

In northern Myanmar and the Myanmar–China border, for example, where the F446I mutation predominates (Huang *et al.*^2015^; Tun *et al.*^2015^; Win *et al.*^2016^), the proportion of infections positive at day 3 was low given the prevalence of K13 mutation and by comparison with observations from the Thailand–Myanmar border and western Cambodia. Using a standard protocol with the assessment of parasitaemia every 6 h following DHA-piperaquine, the parasite clearance half-life in northern Myanmar was 4.7 h, which was significantly shorter than for C580Y studied by the same methodology (Ashley *et al.*^2014^; Tun *et al.*^2016^). Two studies from the China–Myanmar border report results from the RSA in F446I parasites but these come to different conclusions with respect to the *in vitro* phenotype (Wang *et al.*2015b; Ye *et al.*^2016^). Analysis of these isolates indicates that they lack certain elements of the genetic backbone found in much of the rest of Southeast Asia (Miotto *et al.*^2015^; Ye *et al.*^2016^), potentially producing a phenotype that is relatively mild compared to western Cambodia. Transfection-based data would clarify the phenotypic effect of F446I and other mutations.

For these reasons, phenotypic assessments remain an important element of antimalarial susceptibility monitoring studies. It is also possible that alternative mechanisms of resistance will evolve independently of K13.

## SPREAD VERSUS INDEPENDENT EMERGENCE

### Parasite factors

There is little doubt that the K13 mutations associated with artemisinin resistance have emerged *de novo* on multiple occasions (Miotto *et al.*^2013^; Takala-Harrison *et al.*^2015^) producing a ‘soft’ selective sweep (Pennings and Hermisson 2006) across the region (MalariaGEN Plasmodium falciparum Community Project 2016). This genetic observation has been used as an argument to say that ‘firewall’ approaches to containment will not work. But artemisinin resistance as a phenotype has clearly spread substantially over the past decade, and that geographical spread is largely contiguous. This may be explained by spread of the genetic background that favours survival and local spread of resistance mutations (see above).

Furthermore, with the passage of time more resistant mutations take over from less resistant ones and in this manner the ‘soft’ sweep comprising many K13 mutations may be transitioning into a ‘hard’ sweep in which fitter parasites with a dominant K13 mutation take over. In western Cambodia and along the Thailand–Myanmar border, parasites with the C580Y K13 mutation have indeed ‘taken over’, and have also gained the resistance mechanisms to the partner antimalarial drugs. Historical data indicate that pyrimethamine resistance also transitioned from a ‘soft’ phase, with many independent origins of single and double-mutant *dhfr* alleles, into to a ‘hard’ sweep involving triple-mutant *dhfr* from a single origin taking over and spreading across Africa (Roper *et al.*^2004^); again compensations for parasite fitness may have been involved. Stopping the spread of these dominant successful multidrug-resistant parasite genotypes to India and Africa should have the highest international health priority.

### Mosquito factors

*Plasmodium falciparum* has adapted both to humans and anopheline mosquitoes. In the era of malariatherapy, there were clear differences in vector susceptibility between *P. falciparum* isolates from different regions (Shute and Maryon 1954). It is likely that resistant parasites will need to adapt to local vectors and that this may act as a brake on the spread of resistance. While it may slow spread it is unlikely to prevent it—indeed recent evidence shows that artemisinin-resistant parasites from Cambodia can infect *Anopheles gambiae*, the major African malaria vector species, under experimental conditions (St Laurent *et al.*^2015^).

## WHAT CAUSES ARTEMISININ RESISTANCE TO DEVELOP?

Before discussing measures that can be taken to prevent and deal with artemisinin resistance, it is worth reviewing the factors behind its emergence. Southeast Asia has long been considered the epicentre of antimalarial drug resistance; resistance to chloroquine, proguanil, sulphadoxine-pyrimethamine, mefloquine and piperaquine has emerged there. Aside from the obvious long-standing availability of artemisinins in the region, dating back to the 1980s, there is not a wholly satisfactory answer to the question ‘why did resistance start there?’ A wide range of factors related to drug dosing, level of transmission and human behaviour are likely to lead to the emergence of artemisinin resistance (Table 2). Fundamentally, these reflect factors that increase the chance of large numbers of parasites being exposed to drug concentration profiles that are insufficient to eliminate them, with survival and multiplication of the residuum. Pre-treatment hyperparasitaemia is clearly associated with recrudescence (even with artemisinin-sensitive parasites) (WWARN Lumefantrine PK-PD Study Group 2015) both because of the large parasite numbers and the lack of immunity that allowed this large biomass to develop.

**Table 2.** Possible factors promoting artemisinin resistance in Southeast Asia.

Drug administration
Monotherapy
Fake/substandard drugs
Incomplete course
Dosing regimen (artemisinin or partner drug)
Parasite factors
Resistance to partner drug
Hyperparasitaemia
Parasite genetic background
Low transmission
Lower immunity
Single clone infections (less competition)
Higher proportion of symptomatic infections
Host factors
Nutritional state, immunosuppression

Human behaviour is a prominent factor in the development of drug resistance. Malaria in Southeast Asia is largely confined to forested areas, often close to borders, where there is both legal and illegal movement of people, typically young men, who bear the brunt of malaria. These populations often have limited access to health care and commonly self-medicate if they are ill. Substandard or fake (falsified) drugs, artemisinin monotherapies and failure to administer or complete full treatment courses correctly are issues well known in the region (Newton *et al.*^2001^; Yeung *et al.*^2008^; Nayyar *et al.*^2012^; Khin *et al.*^2015^). A single dose of an artemisinin antimalarial probably cannot select resistance that can transmit (White *et al.*^2009^), but repeated administration of non-curative doses can do this. Indeed that is the method employed in the laboratory to select for resistance (Witkowski *et al.*^2010^).

Overall epidemiological context is also critical. In low transmission settings, the human host lacks the immune defence mechanisms that control infections independent of antimalarial drug susceptibility and thus contribute a significant curative effect and reduce substantially the probability that a *de novo* arising resistant mutant parasite will survive to transmit (White 2004; Rogerson, Wijesinghe and Meshnick 2010). Low transmission also means that a higher proportion of infections are symptomatic (leading to a drug encounter) and that there are fewer multiclonal infections and hence less intrahost competition. This increases the chance that drug-resistant parasites with relatively reduced fitness will survive. The propeller region of K13 is highly conserved across the *Plasmodium* genus (MalariaGEN Plasmodium falciparum Community Project 2016) and the mutations observed are hence likely to be associated with functional detriment of some form (Gardner *et al.*^2011^). The low clonality will also tend to preserve retention of supporting loci on other chromosomes rather than separation during meiosis.

Even if correctly taken, current ACT regimens may not contain ideal doses, particularly for vulnerable groups such as children and those with hyperparasitaemia (White *et al.*^2009^). Pharmacokinetic variability means that some patients may not have adequate antimalarial exposure. Such ‘chinks in the armour’ of ACTs may affect the long-term durability of a regimen despite excellent efficacy in short-term therapeutic efficacy studies. In terms of the artemisinin component, the recommended dose of artesunate in artesunate-mefloquine (4 mg kg^−1^) appears necessary for maximal effect. This dose is associated with faster parasite clearance (by 8.1%) compared to 2 mg kg^−1^ in artemisinin-sensitive populations (WWARN Parasite Clearance Study Group 2015). Initial doses of artemisinin derivative vary considerably among different ACTs; artemether-lumefantrine (first dose 1.6 mg kg^−1^ artemether) provided slightly slower clearance than artesunate in the TRAC study (Ashley *et al.*^2014^). Parasite clearance with artemether-lumefantrine was nevertheless similar to that with DHA-piperaquine in a large pooled analysis of clearance rates (based on daily assessment) in Africa (WWARN ACT Africa Baseline Study Group 2015). The DHA dose in DHA-piperaquine is also relatively low (∼2.5 mg kg^−1^).

Pharmacokinetic studies also confirm that dosing regimens do not always provide ideal levels of the partner drug. Piperaquine and lumefantrine dosing in young children have both been suboptimal (WWARN DHA-Piperaquine Study Group 2013; WWARN Artemether-Lumefantrine Dose Impact Study Group 2015; WWARN Lumefantrine PK-PD Study Group 2015). This reflects the use of tablet fractions rather than suspensions, and compounds the effects of lower immunity and sometimes poor nutritional status seen in very young children. The efficacy of loose tablets of artesunate and amodiaquine is inferior to that of the fixed dose coformulation (WWARN Artesunate-Amodiaquine Study Group 2015).

A major problem of increasing contemporary relevance is that ACTs may incorporate partner drugs to which resistance has already arisen, sometimes through cross-resistance; for example, the curative efficacy of artemether-lumefantrine is compromised in infections carrying multiple copies of *pfmdr1* (reflecting long-standing mefloquine usage in Southeast Asia) (Venkatesan *et al.*^2014^). Other genetic factors are also likely to be critical in terms of compensating for the reduced fitness associated with K13 mutations (see above) (Miotto *et al.*^2015^).

## CONCLUSIONS AND FUTURE DIRECTIONS

Most of the human and epidemiological factors behind the development of artemisinin resistance in Southeast Asia will be common to the many areas where *Plasmodium falciparum* malaria transmission is falling, so whether by spread or independent emergence, the potential for artemisinin resistance to affect malaria control on a global scale is obvious. What approaches should be taken to prevent this, or limit its impact?

### Current ACT is not a panacea

While ACTs are clearly preferable to single antimalarials in terms of providing rapidly effective and well-tolerated treatments with prolonged efficacy, a single ACT does not work indefinitely. Extending the course of artemisinin treatment was very effective in the TRAC study in which a 3-day course of artesunate was followed by a standard 3-day ACT (Ashley *et al.*^2014^); an alternative proposal is to give two 3-day ACTs in succession. However, these approaches are unlikely to provide long-lasting improvements in efficacy, and with longer regimens come challenges with adherence.

Until now, the approach taken when an ACT fails has been to switch to another one. But the experience in Cambodia shows that once artemisinin resistance is present, switching ACTs provides only short-term relief. Whether piperaquine resistance arose *de novo* in Cambodia or was imported from China nearly 30 years ago is uncertain, but there is no doubt that for the most part *P. falciparum* parasites were initially sensitive to piperaquine when it was introduced in 2008 in the west of the country. Within a few years parasites had become resistant (Saunders, Vanachayangkul and Lon 2014; Duru *et al.*^2015^), presumably because large numbers were surviving the initial artemisinin phase of treatment, increasing the opportunity for evolution of resistance against slowly cleared piperaquine. Unfortunately, the new ACT artesunate-pyronaridine has a current efficacy of less than 90% in this region (Rueangweerayut *et al.*^2012^; Leang *et al.*^2016^) despite never having been deployed at scale. It is unclear whether this is because the intrinsic activity of pyronaridine does not compensate for the lack of ring-stage killing by artesunate, or because resistance to other drugs causes cross-resistance to pyronaridine. Either way this ACT does not solve the problem.

ACT partner drug resistance should therefore not be considered an independent problem; instead it is simply a natural consequence of underlying artemisinin resistance. The prediction is that ACTs, as a class, will begin to fail across an expanding area of Southeast Asia, and that serial use of standard ACTs will inevitably select resistance to each partner in turn (Boni, White and Baird 2016). Although aiming to help individual patients, using sequential ACTs in an area of established artemisinin resistance is likely to be worse for the community of patients in the long term; despite switching from artesunate-mefloquine to DHA-piperaquine, the cure rate for ACTs in western Cambodia is only worsening.

### The concept of long-term efficacy in communities

In tuberculosis and HIV, at least three drugs are required in the individual patient for cure and viral suppression, respectively. This approach works by raising the probabilistic barrier to resistance. There is a strong argument that malaria should be considered similarly, and that at least three therapeutic challenges need to be applied at the same time to ensure long-term durability of an antimalarial drug regimen (Boni, White and Baird 2016). One way to achieve this is via a triple combination in which two slowly eliminated partner drugs are combined with an artemisinin derivative, retaining the advantage of 3-day treatment. Two such combinations (artemether-lumefantrine-amodiaquine and DHA-piperaquine-mefloquine) are currently being evaluated in multicentre trials examining tolerability, safety and efficacy.

A distinct approach is simultaneous deployment of multiple first-line therapies containing drugs with different or opposing selection pressures, first proposed 30 years ago (Curtis and Otoo 1986). By considering the community as the ‘patient’, multiple first-line therapies could provide a much higher long-term barrier to the development of resistance, and should slow the selection and spread of resistance (Nguyen *et al.*^2015^). Implementation is clearly a challenge but not beyond the scope of national malaria control programmes (Boni, White and Baird 2016), and should be promoted actively.

### New agents

Novel drugs such as the spiroindolones (Rottmann *et al.*^2010^; White *et al.*^2014^) and imidazolopiperazines (Wells, Hooft van Huijsduijnen and Van Voorhis 2015) clearly hold potential to improve efficacy, but they may also be more prone than artemisinins to the development of resistance. Their combination partners are being chosen. The synthetic trioxolanes arterolane (OZ277) and artefenomel (OZ439) share the peroxide pharmacophore of artemisinins and provide broadly the same pharmacodynamic advantage of rapid parasite clearance. Arterolane is already marketed as a combination with piperaquine and provides rapid parasite clearance in children (Toure *et al.*^2015^) and the same curative efficacy as artemether-lumefantrine in a large multicentre study in older individuals (Toure *et al.*^2016^), but it has yet to be tested in patients with artemisinin-resistant infections. Artefenomel is a more stable compound with a longer half-life, offering the possibility of single-dose curative therapy. In a relatively small study at the Thai–Myanmar border, a single dose of artefenomel provided a parasite clearance rate that was slower than that with artesunate on artemisinin-sensitive parasites, but slightly faster than that of artesunate on artemisinin-resistant parasites (Phyo *et al.*2016b). This suggests that cross-resistance with artemisinins might not be extensive, although larger numbers of patients are needed to examine this question, as well as to assess curative efficacy when combined with a suitable partner drug.

### Containment strategy

In response to the clear and present threat posed by artemisinin resistance to global malaria control and hopes for malaria elimination, since 2007 there has been an unprecedented series of meetings, plans and strategy developments accompanied by a substantial increase in donor support for regional containment. These containment efforts largely comprise strengthening of existing malaria control activities. Unfortunately, they have not contained artemisinin resistance which now extends from the coast of Vietnam to the India–Myanmar border. The World Health Organisation has not declared artemisinin resistance a ‘public health emergency of international concern’ as it did for the Ebola virus epidemic in West Africa, or the recent Zika virus epidemic, despite the obvious threat to India and Africa and the lethal precedent where chloroquine resistance (followed by antifol resistance) in *P. falciparum* spread from Southeast Asia to Africa at a cost of millions of lives.

How can resistance be contained in a region where vector control measures are less effective than in other malaria endemic regions and drugs are an essential pillar of malaria control? Controlling malaria by treatment of symptomatic cases alone could distil malaria parasites down to the least drug sensitive—the ‘last man standing is the most resistant’ (Maude *et al.*^2009^). Falciparum malaria will become increasingly difficult to treat. The only way to eliminate resistance is to eliminate falciparum malaria.

### Eliminating falciparum malaria

Elimination of falciparum malaria from the GMS before artemisinin resistance spreads will take a lot more than steady strengthening of conventional malaria control measures. Village health workers (VHW) who diagnose and treat malaria should be present in every village in malaria endemic areas. These workers are the central pillar of effective malaria control, but coverage with effective and well-supported VHWs is still patchy throughout the region. Once VHWs are in place mass treatment with three monthly rounds of DHA-piperaquine and single low dose primaquine, which appears safe even in G6PD-deficient individuals (Bancone *et al.*^2016^), is being piloted in several areas. This is generally effective in reducing malaria in the short term and well tolerated, but it depends critically on the continued efficacy of piperaquine. As piperaquine resistance spreads, the effectiveness of this approach will decline. If adjacent areas are not covered, then malaria will be readily reintroduced. The feasibility and effectiveness of scaling up this approach are also being investigated urgently. The safety, tolerability and effectiveness of mass treatments with drugs other than DHA-piperaquine have not been studied. These and many other questions would ideally be answered before embarking upon a radical elimination campaign, but it is unlikely that we can wait for all relevant research questions to be answered if resistance is to be contained effectively in this dangerous race against time.

## Supplementary Material

Supplementary Data Supplementary data are available at *FEMSRE* online.
