# Supplementary material for: The clinical impact of artemisinin resistance in Southeast Asia and the potential for future spread
Source: FEMS Microbiol Rev. 2017 Jan 1;41(1):34–48. doi: 10.1093/femsre/fuw037 (PMC5424521; doi:10.1093/femsre/fuw037)
Supplement: Supplementary Data — are available at FEMSRE online. [file fuw037_supp.zip › Supplementary_references.docx]

## Additional files:

## References for Figure 1

([Denis*, et al.*, 2002](#_ENREF_9), [Denis*, et al.*, 2006](#_ENREF_10), [Alker*, et al.*, 2007](#_ENREF_1), [Janssens*, et al.*, 2007](#_ENREF_15), [Noedl*, et al.*, 2008](#_ENREF_29), [Dondorp*, et al.*, 2009](#_ENREF_11), [Rogers*, et al.*, 2009](#_ENREF_32), [Noedl*, et al.*, 2010](#_ENREF_30), [Bethell*, et al.*, 2011](#_ENREF_6), [Rueangweerayut*, et al.*, 2012](#_ENREF_33), [Leang*, et al.*, 2013](#_ENREF_17), [Leang*, et al.*, 2013](#_ENREF_18), [Ariey*, et al.*, 2014](#_ENREF_3), [Ashley*, et al.*, 2014](#_ENREF_5), [Lon*, et al.*, 2014](#_ENREF_22), [Leang*, et al.*, 2015](#_ENREF_19), [Spring*, et al.*, 2015](#_ENREF_36), [Amaratunga*, et al.*, 2016](#_ENREF_2), [Leang*, et al.*, 2016](#_ENREF_20)).

## References for Figure 3

([Denis*, et al.*, 2002](#_ENREF_9), [Ashley*, et al.*, 2004](#_ENREF_4), [Mayxay*, et al.*, 2004](#_ENREF_24), [Stohrer*, et al.*, 2004](#_ENREF_37), [Tran*, et al.*, 2004](#_ENREF_43), [Denis*, et al.*, 2006](#_ENREF_10), [Mayxay*, et al.*, 2006](#_ENREF_25), [Vijaykadga*, et al.*, 2006](#_ENREF_45), [Alker*, et al.*, 2007](#_ENREF_1), [Janssens*, et al.*, 2007](#_ENREF_15), [Noedl*, et al.*, 2008](#_ENREF_29), [Dondorp*, et al.*, 2009](#_ENREF_11), [Rogers*, et al.*, 2009](#_ENREF_32), [Thanh*, et al.*, 2009](#_ENREF_40), [Congpuong*, et al.*, 2010](#_ENREF_8), [Mayxay*, et al.*, 2010](#_ENREF_26), [Na-Bangchang*, et al.*, 2010](#_ENREF_28), [Noedl*, et al.*, 2010](#_ENREF_30), [Smithuis*, et al.*, 2010](#_ENREF_35), [Bethell*, et al.*, 2011](#_ENREF_6), [Sun*, et al.*, 2011](#_ENREF_38), [Hien*, et al.*, 2012](#_ENREF_12), [Huang*, et al.*, 2012](#_ENREF_13), [Mayxay*, et al.*, 2012](#_ENREF_27), [Mayxay*, et al.*, 2012](#_ENREF_23), [Rueangweerayut*, et al.*, 2012](#_ENREF_33), [Satimai*, et al.*, 2012](#_ENREF_34), [Thanh*, et al.*, 2012](#_ENREF_41), [Vijaykadga*, et al.*, 2012](#_ENREF_46), [Carrara*, et al.*, 2013](#_ENREF_7), [Kyaw*, et al.*, 2013](#_ENREF_16), [Leang*, et al.*, 2013](#_ENREF_17), [Leang*, et al.*, 2013](#_ENREF_18), [Ariey*, et al.*, 2014](#_ENREF_3), [Ashley*, et al.*, 2014](#_ENREF_5), [Lon*, et al.*, 2014](#_ENREF_22), [Nyunt*, et al.*, 2014](#_ENREF_31), [Takala-Harrison*, et al.*, 2014](#_ENREF_39), [Thriemer*, et al.*, 2014](#_ENREF_42), [Huang*, et al.*, 2015](#_ENREF_14), [Leang*, et al.*, 2015](#_ENREF_19), [Liu*, et al.*, 2015](#_ENREF_21), [Spring*, et al.*, 2015](#_ENREF_36), [Wang*, et al.*, 2015](#_ENREF_47), [Amaratunga*, et al.*, 2016](#_ENREF_2), [Leang*, et al.*, 2016](#_ENREF_20), [Tun*, et al.*, 2016](#_ENREF_44), [Win*, et al.*, 2016](#_ENREF_48))

## References for Figure 4

([Huang*, et al.*, 2012](#_ENREF_13), [Kyaw*, et al.*, 2013](#_ENREF_16), [Ariey*, et al.*, 2014](#_ENREF_3), [Ashley*, et al.*, 2014](#_ENREF_5), [Lon*, et al.*, 2014](#_ENREF_22), [Nyunt*, et al.*, 2014](#_ENREF_31), [Takala-Harrison*, et al.*, 2014](#_ENREF_39), [Thriemer*, et al.*, 2014](#_ENREF_42), [Huang*, et al.*, 2015](#_ENREF_14), [Leang*, et al.*, 2015](#_ENREF_19), [Spring*, et al.*, 2015](#_ENREF_36), [Amaratunga*, et al.*, 2016](#_ENREF_2), [Leang*, et al.*, 2016](#_ENREF_20), [Tun*, et al.*, 2016](#_ENREF_44)).

Alker AP, Lim P, Sem R*, et al.* (2007) Pfmdr1 and in vivo resistance to artesunate-mefloquine in falciparum malaria on the Cambodian-Thai border. *American Journal of Tropical Medicine and Hygiene* **76**: 641-647.

Amaratunga C, Lim P, Suon S*, et al.* (2016) Dihydroartemisinin-piperaquine resistance in Plasmodium falciparum malaria in Cambodia: a multisite prospective cohort study. *Lancet Infect Dis*.

Ariey F, Witkowski B, Amaratunga C*, et al.* (2014) A molecular marker of artemisinin-resistant Plasmodium falciparum malaria. *Nature* **505**: 50-55.

Ashley EA, Krudsood S, Phaiphun L*, et al.* (2004) Randomized, controlled dose-optimization studies of dihydroartemisinin-piperaquine for the treatment of uncomplicated multidrug-resistant falciparum malaria in Thailand. *J Infect Dis* **190**: 1773-1782.

Ashley EA, Dhorda M, Fairhurst RM*, et al.* (2014) Spread of artemisinin resistance in Plasmodium falciparum malaria. *N Engl J Med* **371**: 411-423.

Bethell D, Se Y, Lon C*, et al.* (2011) Artesunate dose escalation for the treatment of uncomplicated malaria in a region of reported artemisinin resistance: a randomized clinical trial. *PLoS One* **6**: e19283.

Carrara VI, Lwin KM, Phyo AP*, et al.* (2013) Malaria burden and artemisinin resistance in the mobile and migrant population on the Thai-Myanmar border, 1999-2011: an observational study. *PLoS Med* **10**: e1001398.

Congpuong K, Bualombai P, Banmairuroi V & Na-Bangchang K (2010) Compliance with a three-day course of artesunate-mefloquine combination and baseline anti-malarial treatment in an area of Thailand with highly multidrug resistant falciparum malaria. *Malar J* **9**: 43.

Denis MB, Davis TM, Hewitt S*, et al.* (2002) Efficacy and safety of dihydroartemisinin-piperaquine (Artekin) in Cambodian children and adults with uncomplicated falciparum malaria. *Clin Infect Dis* **35**: 1469-1476.

Denis MB, Tsuyuoka R, Poravuth Y*, et al.* (2006) Surveillance of the efficacy of artesunate and mefloquine combination for the treatment of uncomplicated falciparum malaria in Cambodia. *Trop Med Int Health* **11**: 1360-1366.

Dondorp AM, Nosten F, Yi P*, et al.* (2009) Artemisinin resistance in Plasmodium falciparum malaria. *N Engl J Med* **361**: 455-467.

Hien TT, Thuy-Nhien NT, Phu NH*, et al.* (2012) In vivo susceptibility of Plasmodium falciparum to artesunate in Binh Phuoc Province, Vietnam. *Malar J* **11**: 355.

Huang F, Tang L, Yang H, Zhou S, Sun X & Liu H (2012) Therapeutic efficacy of artesunate in the treatment of uncomplicated Plasmodium falciparum malaria and anti-malarial, drug-resistance marker polymorphisms in populations near the China-Myanmar border. *Malar J* **11**: 278.

Huang F, Takala-Harrison S, Jacob CG*, et al.* (2015) A Single Mutation in K13 Predominates in Southern China and Is Associated With Delayed Clearance of Plasmodium falciparum Following Artemisinin Treatment. *J Infect Dis*.

Janssens B, van Herp M, Goubert L*, et al.* (2007) A randomized open study to assess the efficacy and tolerability of dihydroartemisinin-piperaquine for the treatment of uncomplicated falciparum malaria in Cambodia. *Trop Med Int Health* **12**: 251-259.

Kyaw MP, Nyunt MH, Chit K*, et al.* (2013) Reduced susceptibility of Plasmodium falciparum to artesunate in southern Myanmar. *PLoS One* **8**: e57689.

Leang R, Barrette A, Bouth DM, Menard D, Abdur R, Duong S & Ringwald P (2013) Efficacy of dihydroartemisinin-piperaquine for treatment of uncomplicated Plasmodium falciparum and Plasmodium vivax in Cambodia, 2008 to 2010. *Antimicrob Agents Chemother* **57**: 818-826.

Leang R, Ros S, Duong S*, et al.* (2013) Therapeutic efficacy of fixed dose artesunate-mefloquine for the treatment of acute, uncomplicated Plasmodium falciparum malaria in Kampong Speu, Cambodia. *Malar J* **12**: 343.

Leang R, Taylor WR, Bouth DM*, et al.* (2015) Evidence of Plasmodium falciparum Malaria Multidrug Resistance to Artemisinin and Piperaquine in Western Cambodia: Dihydroartemisinin-Piperaquine Open-Label Multicenter Clinical Assessment. *Antimicrob Agents Chemother* **59**: 4719-4726.

Leang R, Canavati SE, Khim N*, et al.* (2016) Efficacy and safety of pyronaridine-artesunate for the treatment of uncomplicated Plasmodium falciparum malaria in western Cambodia. *Antimicrob Agents Chemother*.

Liu H, Yang HL, Tang LH*, et al.* (2015) In vivo monitoring of dihydroartemisinin-piperaquine sensitivity in Plasmodium falciparum along the China-Myanmar border of Yunnan Province, China from 2007 to 2013. *Malar J* **14**: 47.

Lon C, Manning JE, Vanachayangkul P*, et al.* (2014) Efficacy of two versus three-day regimens of dihydroartemisinin-piperaquine for uncomplicated malaria in military personnel in northern Cambodia: an open-label randomized trial. *PLoS One* **9**: e93138.

Mayxay M, Khanthavong M, Chanthongthip O*, et al.* (2012) Efficacy of artemether-lumefantrine, the nationally-recommended artemisinin combination for the treatment of uncomplicated falciparum malaria, in southern Laos. *Malar J* **11**: 184.

Mayxay M, Khanthavong M, Lindegardh N*, et al.* (2004) Randomized comparison of chloroquine plus sulfadoxine-pyrimethamine versus artesunate plus mefloquine versus artemether-lumefantrine in the treatment of uncomplicated falciparum malaria in the Lao People's Democratic Republic. *Clin Infect Dis* **39**: 1139-1147.

Mayxay M, Thongpraseuth V, Khanthavong M*, et al.* (2006) An open, randomized comparison of artesunate plus mefloquine vs. dihydroartemisinin-piperaquine for the treatment of uncomplicated Plasmodium falciparum malaria in the Lao People's Democratic Republic (Laos). *Trop Med Int Health* **11**: 1157-1165.

Mayxay M, Keomany S, Khanthavong M*, et al.* (2010) A phase III, randomized, non-inferiority trial to assess the efficacy and safety of dihydroartemisinin-piperaquine in comparison with artesunate-mefloquine in patients with uncomplicated Plasmodium falciparum malaria in southern Laos. *Am J Trop Med Hyg* **83**: 1221-1229.

Mayxay M, Khanthavong M, Chanthongthip O*, et al.* (2012) No evidence for spread of Plasmodium falciparum artemisinin resistance to Savannakhet Province, Southern Laos. *Am J Trop Med Hyg* **86**: 403-408.

Na-Bangchang K, Ruengweerayut R, Mahamad P, Ruengweerayut K & Chaijaroenkul W (2010) Declining in efficacy of a three-day combination regimen of mefloquine-artesunate in a multi-drug resistance area along the Thai-Myanmar border. *Malar J* **9**: 273.

Noedl H, Se Y, Schaecher K, Smith BL, Socheat D, Fukuda MM & Artemisinin Resistance in Cambodia 1 Study C (2008) Evidence of artemisinin-resistant malaria in western Cambodia. *N Engl J Med* **359**: 2619-2620.

Noedl H, Se Y, Sriwichai S*, et al.* (2010) Artemisinin resistance in Cambodia: a clinical trial designed to address an emerging problem in Southeast Asia. *Clin Infect Dis* **51**: e82-89.

Nyunt MH, Hlaing T, Oo HW*, et al.* (2014) Molecular Assessment of Artemisinin Resistance Markers, Polymorphisms in the K13 Propeller, and a Multidrug-Resistance Gene in the Eastern and Western Border Areas of Myanmar. *Clin Infect Dis*.

Rogers WO, Sem R, Tero T*, et al.* (2009) Failure of artesunate-mefloquine combination therapy for uncomplicated Plasmodium falciparum malaria in southern Cambodia. *Malar J* **8**: 10.

Rueangweerayut R, Phyo AP, Uthaisin C*, et al.* (2012) Pyronaridine-artesunate versus mefloquine plus artesunate for malaria. *New England Journal of Medicine* **366**: 1298-1309.

Satimai W, Sudathip P, Vijaykadga S*, et al.* (2012) Artemisinin resistance containment project in Thailand. II: Responses to mefloquine-artesunate combination therapy among falciparum malaria patients in provinces bordering Cambodia. *Malar J* **11**: 300.

Smithuis F, Kyaw MK, Phe O*, et al.* (2010) Effectiveness of five artemisinin combination regimens with or without primaquine in uncomplicated falciparum malaria: an open-label randomised trial. *Lancet Infect Dis* **10**: 673-681.

Spring MD, Lin JT, Manning JE*, et al.* (2015) Dihydroartemisinin-piperaquine failure associated with a triple mutant including kelch13 C580Y in Cambodia: an observational cohort study. *Lancet Infect Dis* **15**: 683-691.

Stohrer JM, Dittrich S, Thongpaseuth V*, et al.* (2004) Therapeutic efficacy of artemether-lumefantrine and artesunate-mefloquine for treatment of uncomplicated Plasmodium falciparum malaria in Luang Namtha Province, Lao People's Democratic Republic. *Trop Med Int Health* **9**: 1175-1183.

Sun XD, Zhang ZX, Wang J*, et al.* (2011) [Therapeutic efficacy and safety of compound dihydroartemisinin/piperaquine for uncomplicated Plasmodium falciparum infection in Laiza City of Myanmar bordering on China]. *Zhongguo Ji Sheng Chong Xue Yu Ji Sheng Chong Bing Za Zhi* **29**: 372-375.

Takala-Harrison S, Jacob CG, Arze C*, et al.* (2014) Independent Emergence of Artemisinin Resistance Mutations Among Plasmodium falciparum in Southeast Asia. *J Infect Dis*.

Thanh NX, Trung TN, Phong NC*, et al.* (2009) Open label randomized comparison of dihydroartemisinin-piperaquine and artesunate-amodiaquine for the treatment of uncomplicated Plasmodium falciparum malaria in central Vietnam. *Trop Med Int Health* **14**: 504-511.

Thanh NX, Trung TN, Phong NC*, et al.* (2012) The efficacy and tolerability of artemisinin-piperaquine (Artequick(R)) versus artesunate-amodiaquine (Coarsucam) for the treatment of uncomplicated Plasmodium falciparum malaria in south-central Vietnam. *Malar J* **11**: 217.

Thriemer K, Hong NV, Rosanas-Urgell A*, et al.* (2014) Delayed parasite clearance after treatment with dihydroartemisinin-piperaquine in Plasmodium falciparum malaria patients in central Vietnam. *Antimicrob Agents Chemother* **58**: 7049-7055.

Tran TH, Dolecek C, Pham PM*, et al.* (2004) Dihydroartemisinin-piperaquine against multidrug-resistant Plasmodium falciparum malaria in Vietnam: randomised clinical trial. *Lancet* **363**: 18-22.

Tun KM, Jeeyapant A, Imwong M*, et al.* (2016) Parasite clearance rates in Upper Myanmar indicate a distinctive artemisinin resistance phenotype: a therapeutic efficacy study. *Malar J* **15**: 185.

Vijaykadga S, Rojanawatsirivej C, Cholpol S, Phoungmanee D, Nakavej A & Wongsrichanalai C (2006) In vivo sensitivity monitoring of mefloquine monotherapy and artesunate-mefloquine combinations for the treatment of uncomplicated falciparum malaria in Thailand in 2003. *Trop Med Int Health* **11**: 211-219.

Vijaykadga S, Alker AP, Satimai W, MacArthur JR, Meshnick SR & Wongsrichanalai C (2012) Delayed Plasmodium falciparum clearance following artesunate-mefloquine combination therapy in Thailand, 1997-2007. *Malar J* **11**: 296.

Wang Y, Yang Z, Yuan L*, et al.* (2015) Clinical Efficacy of Dihydroartemisinin-Piperaquine for the Treatment of Uncomplicated Plasmodium falciparum Malaria at the China-Myanmar Border. *Am J Trop Med Hyg* **93**: 577-583.

Win AA, Imwong M, Kyaw MP, Woodrow CJ, Chotivanich K, Hanboonkunupakarn B & Pukrittakayamee S (2016) K13 mutations and pfmdr1copy number variation in Plasmodium falciparum malaria in Myanmar. *Malaria Journal* **15**.
